# Survival of dental implants and occurrence of osteoradionecrosis in irradiated head and neck cancer patients: a systematic review and meta-analysis

**DOI:** 10.1007/s00784-021-04065-6

**Published:** 2021-08-16

**Authors:** Daniel Jan Toneatti, Ronny Roger Graf, John-Patrik Burkhard, Benoît Schaller

**Affiliations:** Department of Cranio-Maxillofacial Surgery, Inselspital, Bern University Hospital, University of Bern, CH-3010 Bern, Switzerland

**Keywords:** Radiotherapy, Dental implants, Osteoradionecrosis, Mandibular reconstruction, Bone grafting, Hyperbaric oxygenation

## Abstract

**Objectives:**

This systematic review assesses dental implant survival, calculates the incidence rate of osteoradionecrosis, and evaluates risk factors in irradiated head and neck cancer patients.

**Materials and methods:**

Various databases (e.g., Medline/Embase using Ovid) and gray literature platforms were searched using a combination of keywords and subject headings. When appropriate, meta-analysis was carried out using a random effects model. Otherwise, pooled analysis was applied.

**Results:**

A total of 425 of the 660 included patients received radiotherapy. In total, 2602 dental implants were placed, and 1637 were placed in irradiated patients. Implant survival after an average follow-up of 37.7 months was 97% (5% confidence interval, CI 95.2%, 95% CI 98.3%) in nonirradiated patients and 91.9% (5% CI 87.7%, 95% CI: 95.3%) after an average follow-up of 39.8 months in irradiated patients. Osteoradionecrosis occurred in 11 cases, leading to an incidence of 3% (5% CI 1.6%, 95% CI 4.9%). The main factors impacting implant survival were radiation and grafting status, while factors influencing osteoradionecrosis could not be determined using meta-analysis.

**Conclusion:**

Our data show that implant survival in irradiated patients is lower than in nonirradiated patients, and osteoradionecrosis is—while rare—a serious complication that any OMF surgeon should be prepared for. The key to success could be a standardized patient selection and therapy to improve the standard of care, reduce risks and shorten treatment time.

**Clinical relevance:**

Our analysis provides further evidence that implant placement is a feasible treatment option in irradiated head and neck cancer patients with diminished oral function and good long-term cancer prognosis.

## Introduction

Oral and pharyngeal malignancies are the sixth most common cancers worldwide and comprise approximately 3.6% of all cancers [1, 2]. Approximately 90% of these malignancies are squamous cell carcinomas, which typically occur in males over 50 years old [3]. Tobacco and alcohol consumption alone increase the risk for oral squamous cell carcinoma by factors of 3.43 and 2.54, respectively [4, 5]. Combining these two shows a more than multiplicative synergistic risk increase [6]. Oral squamous cell carcinoma is also often associated with poor oral hygiene [7].

Patients with oral and pharyngeal cancer treated with ablative surgery and adjuvant radiotherapy have a 5-year survival rate of 51.3%. Unfortunately, this rate has been stable over the last decades and cannot be significantly improved because detection is still delayed [8].

Cancer treatment results in significant morbidity and oral rehabilitation that is often unsatisfactory for patients [9]. Their main communication feature—their face—has changed. Their speech, mastication, swallowing, and breathing may be severely compromised, and their oral comfort, facial expression, and esthetics may be impacted. Consequently, their psychological condition, social life, and quality of life in general might be diminished [10].

Some of these challenges could be resolved by using dental implants during oral rehabilitation. However, many clinicians view implants as contraindicated and are currently reluctant to use them in irradiated head and neck cancer patients [11]. Their main concerns are altered anatomy and impaired wound healing, which make placing implants in the correct prosthodontic position difficult, and complications such as failed osseointegration, soft tissue hyperplasia, and osteoradionecrosis (ORN) more probable.

### Objective of this review

The main goal of this systematic review was to study the occurrence of osteoradionecrosis after placing dental implants in irradiated jaws by calculating an incidence rate and isolating risk factors. At the same time, the extracted data were used to determine a survival rate for dental implants in irradiated patients.

The target questions were “What is the survival rate of dental implants in irradiated patients?”, “How common is ORN after dental implant surgery in irradiated patients?” and “Which factors influence implant survival and development of ORN in irradiated head and neck cancer patients?”.

Answering those questions is clinically significant as they would allow the selection of patients with a low risk and the application of beneficial preventive methods in patients with a high risk for implant failure and ORN.

## Materials and methods

This review followed the Preferred Reporting Items for Systematic reviews and Meta-Analyses (PRISMA) and the Meta-Analysis Of Observational Studies in Epidemiology (MOOSE) statement guidelines [12, 13]. Additionally, the Cochrane handbook was used [14].

The full study protocol can be assessed on the International Prospective Register of Systematic Reviews (PROSPERO, identification code: CRD42018107153) or using Mendeley Data [15].

### Search strategy

Initially, a preliminary search on Google Scholar, Microsoft Academic, and PROSPERO was performed to identify core articles and to estimate the need for a systematic review.

Because the examiners concluded that there was a lack of evidence, an electronic search was set up. The universal “Patient, Intervention, Control, Outcome” (PICO) and the more specific “Condition, Context, Population” (CoCoPop) method were applied and used as a framework to develop a search strategy in which keywords and subject headings were combined [16, 17].

The electronic search strategy was reviewed by the co-examiners using the Peer Review of Electronic Search Strategies (PRESS) checklist [18].

Electronic searches (up until 05.04.2021) were performed on multiple databases, including Ovid (MEDLINE, Embase), PubMed, CENTRAL, CINAHL, PsycINFO, Scopus, and Web of Science. Databases for gray literature were selected using the Canadian Agency for Drugs and Technologies in Health (CADTH) “Grey matters checklist” and the guidelines of the University of Bern [19, 20]. To limit the overlap of results between Ovid and PubMed, a filter proposed by the CADTH was applied [21]. Otherwise, no filters or limitations were utilized.

Additionally, a manual search was carried out using core articles and reviews, which came up during the search process.

The PICO and CoCoPop frameworks and the complete search strategy used on the Ovid database can be found in Mendeley Data [15].

### Eligibility criteria

Eligibility criteria were created following the approach described by Munn et al. (2015) and the Cochrane handbook [14, 22]. The PICO and CoCoPop framework served as a foundation for creating those inclusion/exclusion criteria.

An article was included if its full text was available in English, French, or German. Published and gray literature were regarded equally. The studies had to contain clinical data, either obtained in an observational or interventional study design. Case reports, reviews, guidelines, or data collected in a survey were excluded.

The study sample had to consist of a minimum of 5 patients, who each underwent radiotherapy in the head and neck region (affecting mandible, maxilla, or both) and had an edentulous area that needed oral rehabilitation using at least one dental implant. Studies containing data on nonorally used facial implants were excluded.

Articles including patients undergoing comedications or cointerventions (e.g., additional chemotherapy, reconstructive surgery, hyperbaric oxygen therapy) were allowed, and those procedures were specifically recorded.

Eligible studies had to report on essential data (number of patients, number of implants placed, number of failed implants, reason for implant failures, minimum duration of follow-up), and if subgroups (e.g., nonirradiated control) were present, the division had to be reproducible and essential data had to be assignable to each group individually.

If a case of osteoradionecrosis was diagnosed, there needed to be a topological and chronological correlation between the placed implant and the occurrence of ORN.

The minimum follow-up for individual subjects was at least 3 months and for groups at least 6 months after implantation. Studies with unclear minimum follow-up durations were excluded.

### Study selection

The obtained search results were exported to Mendeley (Version 1.19.4). First, only the study title and abstract were considered for study selection. Potentially included studies were further assessed using their full text. The eligibility criteria determined which studies were included in this review (for the title/abstract and full text analysis), and for every study, the reason for exclusion was recorded in Mendeley.

**Fig. 1:**
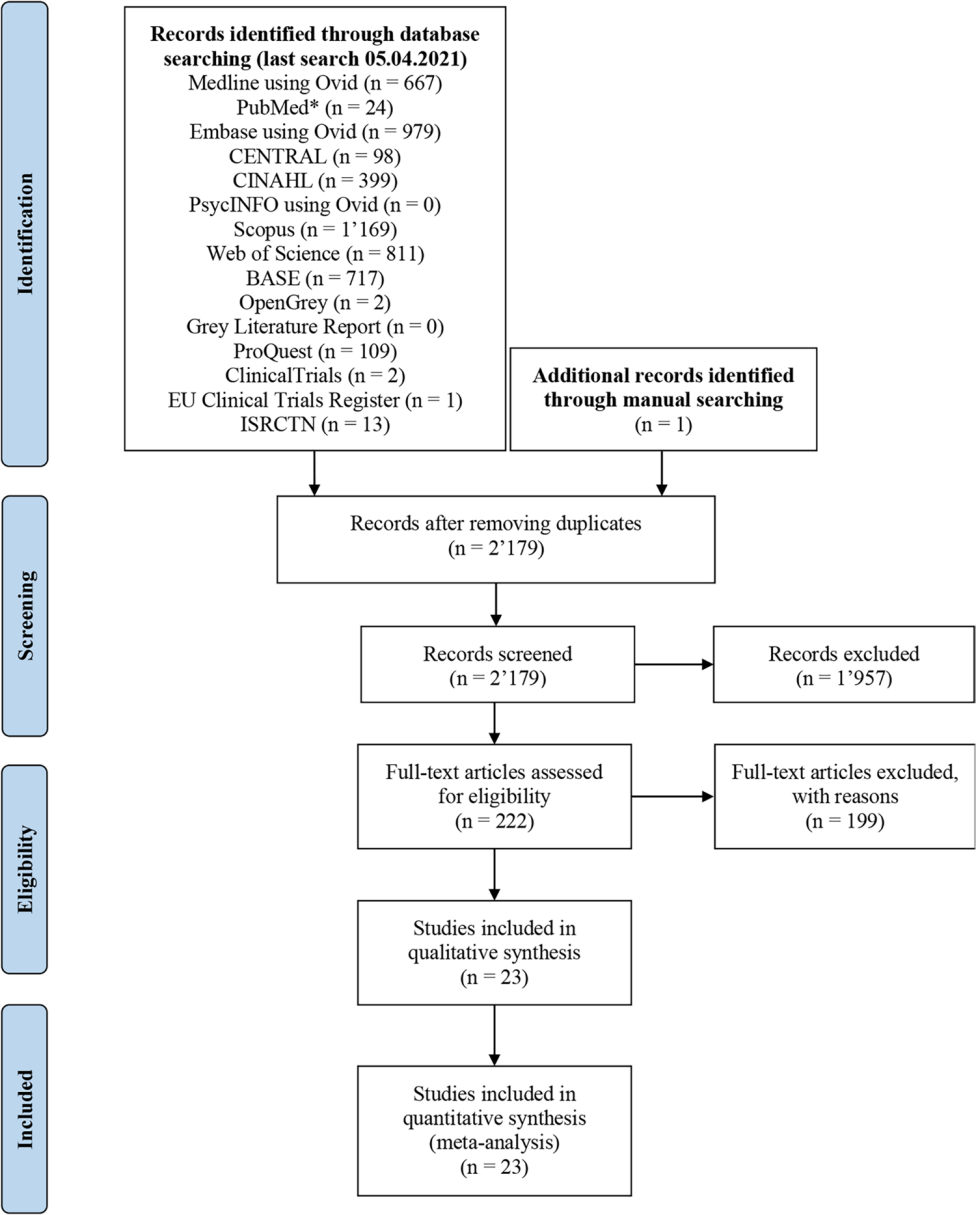
Adapted PRISMA flow-diagram. A single asterisk (^*^) indicates application of a filter proposed by the CADTH

The study selection process can be tracked in detail in the PRISMA flow diagram in Fig. 1.

This procedure was done by a single individual. However, doubtful cases and the included studies were all discussed with the co-examiners.

### Data extraction

The following data extraction was performed in Microsoft® Excel® using the full text of the included studies. If possible, subgroups were separated, and missing data for those subgroups were calculated if a detailed patient demographics or implant survival table was available.

### Quality assessment

The limitations of each article were assessed using a checklist from The Joanna Briggs Institute that was appropriate for the respective study design [23].

### Statistical analysis

The descriptive statistics were performed using the built-in Microsoft® Excel® tools. For meta-analysis, a random effects model was introduced. Incidence data were handled as proposed by Barendregt et al. (2013) using a double arcsine transformation in MetaXL (Version 5.3) [24, 25]. Subgroups were analyzed in RevMan (Version 5.4) [26]. If meta-analysis was not possible, pooled analysis was used.

It is important to point out that no article was excluded during analysis due to its results. However, some studies could not be included in the calculation due to the lack of specific data (e.g., average age, sex distribution) or due to mixed subgroups (e.g., occurrence of osteoradionecrosis in grafted compared to nongrafted bone).

Other studies labeled implants in deceased patients or in patients with tumor recurrences as failures. The same can be said about implants who were “put to sleep.” Since this would lead to false low survival rates, these data were corrected by excluding the implants in question in implant survival analysis and during pooled analysis.

## Results

A summarized table of the extracted data can be found in Tables 1, 2, and 3. The full data extraction, a complete quality assessment and the forest and funnel plots corresponding to the results below can be accessed on Mendeley data [15].

**Table 1:** Summary of extracted data

Author	Study typ e (POS, ROS, RCT)	Number of pt	Age (years)	Gender		Mean radiation dosage (gray)	HBO	Impl location (number of impl)		Impl plac ed	Mean time interval		Impl in sit u	Impl los t	ORN cas es	Mean follow-up (months)
				Male	Female			max vs. mand	Native vs. graft		RT and impl (months)	Impl and loading (months)				
Barber et al. 1995 [80]	ROS	5	Ø	Ø		> 50	5	mand	Graft	20	> 2, post-RT	6	20	0	0	Ø
Franzén et al. 1995 [81]	ROS	5	Ø	2	3	40.3	0	mand	Ø	20	24, post-RT	3.5–6 M + 10–14 T	19	1	0	Ø
Watzinger et al. 1996 [82]	ROS	26 (total)	62	21	5	50	Ø	mand	Mixed	136	12, post-RT	> 6	94	42	1	Ø
		Ø	Ø	Ø		50	Ø	mand	Native	60	12, post-RT	> 6	46	14	0	Ø
		Ø	Ø	Ø		50	Ø	mand	Native	24	12, post-RT	> 6	15	9	1	Ø
		Ø	Ø	Ø		50	Ø	mand	Graft	52	12, post-RT	> 6	33	19	0	Ø
Weischer et al. 1996 [83]	ROS	14	57	3	11	0	0	mand	Native: 22 Graft: 26	48	Ø	3–6	45	3	0	Ø
		13	56	5	8	42.5	0	mand	Native: 36 Graft: 21	57	> 13, post-RT	> 6	53	4	0	Ø
Keller et al. 1997 [84]	ROS	19 (total)	60	9	10	Ø	Ø	mand	Native: 72 Graft: 26	98	Ø	> 4	87	11	0	Ø
		18	60	8	10	56 (2 pt Ø)	Ø	mand	Native: 72 Graft: 22	94	72, post-RT	> 4	83	11	0	Ø
		1	53	1	0	60	Ø	mand	Graft	4	10, pre-RT	4	4	0	0	Ø
Weischer et al. 1997 [85]	ROS	8	55	7	1	0	Ø	mand	Native	30	Ø	3	27	3	0	38
		12	55	9	3	0	Ø	mand	Graft	51	Ø	3–6	49	2	0	39
		9	54	8	1	44	Ø	mand	Native	43	48, post-RT	6	38	5	0	36
		8	56	7	1	44	Ø	mand	Graft	33	48, post-RT	6	28	5	0	36
Andersson et al. 1998 [86]	ROS	15	68	11	4	54.3	0	max: 12 mand: 78	Native	90	22.1, post-RT	max.: 6 mand.: 3–6	88	2	0	27.8
August et al. 1998 [87]	ROS	1	72	1	0	0	0	Ø		2	Ø	4	2	0	0	9
		17	63.8	9	8	62.3	2	Ø		38	44.5, post-RT	3.6	38	0	0	17.4

*HBO*, hyperbaric oxygen therapy; *impl*, implants; *mand.*, mandible; *max*, maxilla; *ORN*, osteoradionecrosis; *POS*, prospective observational study; *pt*, patients; *RCT*, randomized control study; *ROS*, retrospective observational study; *RT*, radiotherapy; *Ø*, unspecified

**Table 2:** Summary of extracted data

Author	Study type (POS, ROS, RCT)	Number of pt	Age (ye ars)	Gender		Mean radiation dosag e (gray)	HBO	Impl location (number of impl)		Impl placed	Mean time interval		Impl in sit u	Impl lost	ORN cas es	Mean follow-up (months)
				Male	Female			max vs. mand	Native vs. graft		RT and impl (months)	Impl and loading (months)				
Brogniez et al. 1998 [88]	ROS	19	53	16	3	57 (1 pt Ø)	0	max: 3, mand: 50	Mixed	53	17, post-RT	6	36	17	0	38
Keller et al. 1998 [89]	ROS	31 (total)	50.5	16	15	Ø	0	mand	Mixed	154	Ø	Ø	147	7	0	64.2
		19	41.5	12	7	0	0	mand	Native: 19 Graft: 72	91	Ø	7.8	85	6	0	77.5
		3	41.3	1	2	0	0	mand	Native: 7 Graft: 9	16	Ø	5–6	16	0	0	28.7
		4	65.5	0	4	62.1	0	mand	Native: 12 Graft: 10	22	> 7, post-RT	4–6	22	0	0	50
		5	57	3	2	60.8	0	mand	Native: 7 Graft: 18	25	> 13, post-RT	4–6	24	1	0	42.2
Esser et al. 1999 [90]	ROS	28	55.3	23	5	0	0	mand	Native	128	Ø	5.1	106	22	0	53.3
		34	54.7	28	6	60	0	mand	Native	148	23.1, post-RT	5.2	73	75	1	62.2
Schoen et al. 2003 [91]	ROS	5	54	3	2	61.6	Ø	mand	Ø	20	1.5, pre-RT	6	16	4	0	22
Iizuka et al. 2005 [76]	ROS	28 (total)	58.2	19	9	65: 19 pt 0: 9 pt	0	mand	Ø	37	Ø	Ø	37	0	0	45
		18	Ø	Ø		65: 9 pt 0: 9 pt	0	mand	Ø	24	Post-RT: 9 pt	Ø	24	0	0	Ø
		10	Ø	Ø		65	0	mand	Ø	13	Pre-RT: 7 pt Pre + post-RT: 3 pt	Ø	13	0	0	Ø
Shaw et al. 2005 [92]	ROS	77 (total, 4 dropouts excluded)	58	49	32	Ø	24	Mixed	Native: 241 Graft: 123	364	Ø	3–6	308	56	2	42
		43	Ø	Ø		Ø	7	Mixed	Ø	192	Ø	3–6	167	25	0	Ø
		34	Ø	Ø		50 (3 pt Ø)	17	Mixed	Ø	172	12, post-RT	3–6	141	31	2	Ø

*HBO*, hyperbaric oxygen therapy; *impl*, implants; *mand.*, mandible; *max*, maxilla; *ORN*, osteoradionecrosis; *POS*, prospective observational study; *pt*, patients; *RCT*, randomized control study; *ROS*, retrospective observational study; *RT*, radiotherapy; *Ø*, unspecified

**Table 3:** Summary of extracted data

Author	Study type (POS, ROS, RCT)	Number of pt	Age (years)	Gender		Mean radia tion dosage (gray)	HBO	Impl location (number of impl)		Impl placed	Mean time interval		Impl in situ	Impl lost	ORN cases	Mean follow-up (msonths)
				Male	Female			max vs. mand	Native vs. graft		RT and impl (months)	Impl and loading (months)				
Bodard et al. 2006 [93]	ROS	33	60.3	32	1	60.5	0	max: 6 mand: 62	Native	68	54.2, Post-RT	7	68	0	0	31.9
Landes et al. 2006 [47]	ROS	30 (total)	63	22	8	Ø	Ø	mand	Native	114	Ø	25 days	113	1	0	36
		11	Ø	Ø		0	Ø	mand	Native	42	Ø	25 days	42	0	0	Ø
		19	Ø	Ø		57	Ø	mand	Native	72	21, Post-RT	25 days	71	1	0	Ø
Schoen PJ et al. 2007 [94]	RCT	13	61.2	8	5	64.5	0	mand	Native	49	48, Post-RT	6	46	3	0	Ø
		13	57.7	9	4	60.5	13	mand	Native	54	45, Post-RT	6	46	8	1	Ø
Liberali 2009 [95]	POS (Non-RCT)	14	59.6	13	1	61.57	14	mand	Ø	28	67.7, post-RT	6.5	25	3	1 (+ 1 after implant failure) (+ 1 unrelated to implant site)	Ø
Fenlon et al. 2012 [59]	ROS	29	Ø	Ø		0	Ø	mand	Graft	110	Ø	Ø	107	3	0	Ø
		12	Ø	Ø		65	Ø	mand	Graft	35	Pre-RT	6	20	15	3	Ø
Linsen et al. 2012 [96]	ROS	66	55.7	43	23	Ø	0	max: 49 mand: 213	Native: 183 graft: 79	262	41, post-RT	4.9	248	14	0	47.99
		32	Ø	Ø		0	0	max: 36 mand: 103	native: 95 graft: 44	139	Ø	4.9	133	6	0	Ø
		34	Ø	Ø		60: 8 pt 36: 26 pt	0	max: 13 mand: 110	native: 42 graft: 81	123	41, post-RT	4.9	115	8	0	Ø
Curi et al. 2018 [97]	ROS	35	65	18	17	62	13	max: 79 mand: 90	Ø	169	23.7, post-RT	6	157	12	0	89.2
Desoutter et al. 2018 [98]	ROS	18	57.5	14	4	51.8	Ø	mand	native	40	44.6, post-RT	2–4	32	8	(+ 1 after implant failure)	Ø
Takahashi et al. 2018 [99]	ROS	23 (total)	71.4	11	12	Ø	Ø	max: 54 mand: 49	native: 80 graft: 23	103	Ø		91	12	0	64.4
		6	Ø	Ø		59	Ø	Ø		11	Ø		5	6	0	Ø

*HBO*, hyperbaric oxygen therapy; *impl*, implants; *mand.*, mandible; *max*, maxilla; *ORN*, osteoradionecrosis; *POS,* prospective observational study; *pt*, patients; *RCT*, randomized control study; *ROS*, retrospective observational study; *RT*, radiotherapy; *Ø*, unspecified

In this systematic review, the data of 23 studies, with an average of 28.7 patients each, were extracted. This accumulates to information for approximately 660 patients. Of those, 63% (416 patients) were male, 31% (202 patients) were female, and in 6.3% (42 patients), sex was not specified. The average age was 59.5 (± 5.4) years. A total of 638 patients underwent head and neck cancer treatment, while the other 22 noncancer patients were otherwise surgically reconstructed.

Most patients (63%, 414 patients) in the included articles suffered from squamous cell carcinoma. A total of 425 (64.4%) of the 660 patients were treated with radiotherapy and received an average radiation dose of 55.8 (± 7.6) Gray. Chemotherapy was given to 12.4% (82 patients).

In total, 2602 dental implants were placed, 1637 (62.9%) of which were in the irradiated subgroup. This leads to an average of 3.9 dental implants per patient. The most frequently used location for dental implants was the mandible (1995 implants or 76.7%). The maxilla was used for 203 implants (7.8%), and in 404 implants (15.5%), the implant location remained unclear. In 3 studies (68 implants in 27 irradiated patients), implants were placed primarily, while in the other 20 reports (1702 in at least 416 irradiated patients), secondary implant placement was used. In the study of Watzinger et al. (1996), a subgroup with an unknown size underwent 52 partly immediate, partly delayed implantations.

The average interval between primarily placed implants and radiotherapy was 6 weeks, whereas in the case of secondary implant surgery, a median waiting period of 30.7 months was respected. During implantation, antibiotics were given to 42.1% (179 irradiated patients), and hyperbaric oxygen therapy was used in 15.1% of irradiated patients (64 irradiated patients).

After an average follow-up of 37.7 months, 97% (5% confidence interval; CI 95.2%; 95% CI 98.3%) of implants were still in situ in the nonirradiated control group, whereas the irradiated subgroup had a survival rate of 91.9% (5% CI 87.7%; 95% CI; 95.3%) after an average 39.8-month follow-up.

Meta-analysis for implant survival confirmed that radiotherapy is a risk factor for implant survival (OR 2.68 resp. 3.08). However, pooled analysis showed that an average radiation dose above 60 Gray did not seem to negatively influence implant outcome (*p*-value 0.89). A waiting period of more than 12 months between irradiation and implantation seems to be beneficial (OR 3.2), and the application of hyperbaric oxygen before implant placement had a nonsignificant impact (*p*-value 0.17 resp. 0.98) in pooled analysis.

Placing implants in grafted bone showed a significantly worse prognosis only in pooled analysis of irradiated patients (OR 2.67). In nonirradiated patients, implants inserted in native and grafted bone showed a comparable clinical outcome (*p*-value 0.29 resp. 0.93).

Pooled analysis also suggests that primary/immediate implant surgery increases the risk of implant failure compared to using a secondary/delayed procedure (OR 3.2). No difference between submerged and nonsubmerged surgical approaches (*p*-value 0.19) in irradiated patients was noted. Loading implants before 6 months seemed to increase the number of implants in situ (OR 0.56).

Osteoradionecrosis occurred in 11 cases, leading to an incidence of 3% (5% CI 1.6%; 95% CI 4.9%). Due to this complication, an average of 2.5 implants were lost. Two of the 11 cases occurred after implant failure. One additional event was unrelated to the implant site and therefore not included in the calculations.

Pooled analysis showed that the risk for ORN was unrelated to the radiation dose surpassing 60 Gray (*p*-value 0.37) or if a waiting period of 12 months between radiotherapy and implantation was respected (*p*-value 0.1). Patients undergoing hyperbaric oxygen therapy had an increased risk for osteoradionecrosis (OR 5.95). Implant surgery involving grafted bone demonstrated a higher number of cases compared to native bone (OR 6.8). Immediate/primary implantation appeared to increase risk (OR 7.3) in this group of patients, while no difference between submerged and nonsubmerged approaches could be detected (*p*-value 0.99). Implant loading protocols (earlier or later than 6 months after implantation) also did not seem to influence ORN occurrence (*p* value 0.37).

## Discussion

The main objective of this systematic review was to investigate the benefits and risks of dental implantation in irradiated head and neck cancer patients and to study the occurrence of osteoradionecrosis after this surgical procedure.

Three focus questions were developed: “What is the survival rate of dental implants in irradiated patients?”, “How common is osteoradionecrosis after dental implant surgery in irradiated patients?” and “Which factors influence implant survival and development of osteoradionecrosis in irradiated head and neck cancer patients?”.

The mostly male patients (63%, 416 patients) and those with median age of 59.5 years were at high risk for oral malignancies. A multicenter study by Dhanuthai et al. (2017) including 6151 oral cancer patients showed a gender distribution of 68.9% males and a mean age of 58.37 years [27]. Tandon et al. (2017) published comparable results in their 10-year retrospective study (age peak above 50 years, 59.01% males) [3]. The higher prevalence in men can be explained by their more common risk behaviors.

Sixty-three percent (414 patients) in this systematic review suffered from oral squamous cell carcinoma. This is lower than 90%, which could be expected for cancers of the oral cavity [3]. This difference could be caused by the high number of patients (27.4%, 181 patients) in which the type of pathology was not clearly specified.

### Implant survival

The calculated implant survival during the average 37.7-month follow-up in the nonirradiated control group was high (97%) but still considerably lower than the 10-year survival rate reported by Buser et al. in 2012 (98.8%) [28]. However, the control group mostly consisted of head and neck cancer patients in which ablative surgery was necessary. This makes altered—sometimes even reconstructed—anatomy common and therefore proper implant positioning, successful osseointegration, and complication-free soft tissue healing even without radiotherapy harder to achieve. Suboptimal implant positioning could lead to occlusal overloading, while soft tissue hyperplasia could make plaque control more difficult. Patients affected by oral carcinomas often have a history of alcohol and/or tobacco abuse and show worse oral hygiene [4, 5, 7]. Additionally, those patients suffer from increased morbidity, again negatively impacting their capability for sufficient plaque control. In contrast to the Bernese study in which only nonrisk patients were treated with Straumann® SLA® implants (Straumann® Dental Implant System, Straumann Holding AG, Basel, Switzerland) and most implants were placed by a single surgeon, this systematic review—by nature—consists of data in which different implant types and manufacturers were used by various surgeons. All these factors could have negatively impacted implant survival.

In the irradiated group, the implant survival during the average 39.8-month follow-up was lower (91.9%) than that in the control group. This was expected as those patients have an even higher morbidity (e.g., trismus, fibrinoid soft tissue erosions or xerostomia), impacting their capability for proper oral hygiene. Furthermore, irradiated tissue shows worse wound healing, and a risk for the development of osteoradionecrosis exists. These survival rates are in agreement with the one obtained by Smith Nobrega et al. (2016) at 84.3% after up to 192 months [29].

Excluding patient death or tumor recurrence, most implants in the control and irradiated groups were lost due to failed osseointegration (42.9%), followed by peri-implantitis (28.6%). Other reasons included pathological bone fractures, occlusal overloading, excessive soft tissue hyperplasia, soft tissue necrosis, or osteoradionecrosis. Presumably, occlusal overloading is caused by implant malposition as bite force is on average 50% lower in resected jaws than in healthy controls [30].

The lower implant survival in irradiated patients could be reproduced during meta-analysis. In contrast to other authors—who established an increased risk for implant failure if the applied dosage surpassed 50–60 Gray—we were unable to prove the existence of a radiation dose-failure relationship [31–33].

Introducing subgroups depending on the patient grafting status lowered the heterogeneity of the sample. Further pooled analysis showed that placing implants in grafted bone seems to negatively influence implant survival (OR 2.67). In the meta-analysis of Schiegnitz et al. (2014), an odds ratio of 1.82 for implant placement in grafted bone was calculated [34]. Shugaa-Addin et al. (2016) also showed a lower survival of implants in grafted than native bone. In particular, nonvascularized bone grafts seem to be associated with lower implant survival. According to this study, bone grafts show a lower bone density, volume, vascularization, and higher resorption [35].

Our pooled data suggest that placing implants during initial surgery increases the risk for implant failure (OR 3.2). A recent review by Alberga et al. (2020) came to the opposite conclusion [36]. As shown by Schoen et al. (2008), neither immediate nor delayed implantation is ideal [37]. Hence, it could be useful to adapt the approach to the individual clinical situation and the available resources.

A relevant impact on implant survival depending on the interval between radiotherapy and implant placement could also be detected in pooled analysis. In our study, placing implants during the first 12 months after irradiation increased the risk for implant failure (OR 3.2). Claudy et al. (2015), who showed similar results and provided data, showed that placement of dental implants between 6 and 12 months after radiotherapy was associated with a 34% higher risk of failure [38]. Some argue that bone recovery occurs 6–12 months after radiotherapy, which could improve the patient’s regeneration capacity [39]. This is verified by a higher necessary removal torque [40]. In contrast, Chrcanovic et al. (2016) were unable to show a significant improvement after a waiting period of more than 1 year [41]. Granström et al. (2006) proposed keeping the interval rather short because over time (> 10 years), progressive endarteritis could reduce the healing potential [42]. This leads to the conclusion that time has two antagonistic effects on irradiated tissue recovery. There is a short-term positive cellular effect improving the bone healing capacity and a long-term negative effect on vascularity [31]. Another aspect to consider is tumor recurrence, which often occurs within the first year. Therefore, while waiting for 12 months could lead to some benefits—such as better tissue healing and increased tumor control—additional surgical delay might not be indicated, and a patient’s risk for complications might even increase.

If a significant difference in implant survival between the maxilla and mandible or anterior and posterior implant position exists, could not be analyzed due to insufficient data. However, in 1996, Eckert et al. already proved implantation in the mandible to be more successful [43]. Systematic reviews by Chambrone et al. (2013) and Colella et al. (2007) on dental implants in irradiated patients were able to verify those results by showing a higher survival rate in the mandible [44, 45]. This could be caused by the higher amount of dense, compact bone, which leads to better primary stability. Data from Lee et al. (2012) suggest that the anterior mandible is often spared from high radiation doses during radiotherapy, which could benefit anterior implant placement [46].

In the included study by Landes et al. (2006), implants were placed in a one-stage procedure, and prosthodontic rehabilitation took place according to early-loading principles [47]. Although those nonsubmerged implants did not show a reduced survival rate, further studies—preferably with a prospective design—are needed to endorse this approach. However, according to our data, the waiting period of 6 months as endorsed by many does not seem to be essential in providing optimal care.

A systematic review by Shah et al. (2017) on hyperbaric oxygen therapy (HBO) showed a reduced risk for implant failure (reducing the risk by a factor of 1.2), while a Cochrane review by Esposito et al. (2013) did not recommend its use due to the lack of randomized controlled trials [48, 49]. A recent randomized controlled trial by Shaw et al. (2019) supports our data that HBO does not seem to significantly improve implant survival [50].

Other potential risk factors for implant loss in irradiated jaws, such as age, smoking status, chemotherapy, or use of antibiotics, could not be isolated in the present study due to insufficient reporting. Moy et al.’s (2005) data indicate a higher risk for implant failure in above 60-year-olds, smokers, or patients suffering from diabetes. Neither Moy et al. (2005) nor Kovács (2001) demonstrated reduced implant survival after chemotherapy [51, 52]. Unfortunately, there is no evidence on the effectiveness of prescribing antibiotics before, during, or after implant surgery in irradiated patients [53]. Furthermore, we want to emphasize the importance of proper oral hygiene, adequate prosthetic rehabilitation and frequent follow-up appointments.

It is also notable that implant loss in most cases does not lead to complete rehabilitation failure [54].

### Osteoradionecrosis

In their 30-year retrospective review, Reuther et al. (2003) reported an overall osteoradionecrosis incidence rate of 8.2% [55]. After tooth extraction, Nabil et al. (2011) described a 7% incidence rate if no preventive method was used, while a lower incidence rate could be observed after applying hyperbaric oxygen (4%) or antibiotics (6%) [56].

Few studies estimate the incidence of ORN after implant surgery. Wagner et al. (1998) stated an incidence of 1.6% (1 case, 63 irradiated patients), and Keller et al.’s (1997) review proposed an incidence of 1.8% (3 cases, 170 irradiated patients) [57, 58]. In our study, osteoradionecrosis occurred in 11 out of 425 irradiated patients after implantation, resulting in an incidence rate of 3%. While our proposed incidence rate is higher than those previously published, those values are still inside our calculated confidence intervals (5% CI 1.6%; 95% CI 4.9%). A possible explanation for this difference is the uncommonness of osteoradionecrosis. Hence, inclusion of a single study with a high number of cases could impact the estimated incidence. In our study, Fenlon et al. (2012) provided 3 out of 11 ORN cases [59]. The small sample size of the previously published articles could also play a role.

Because many of the physiological aspects of irradiated bone and the molecular factors that lead to osteoradionecrosis remain unclear, isolating risk factors and finding efficient preventive methods is difficult. The rare and unpredictable appearance of osteoradionecrosis further aggravates this problem. The following subgroup analysis should thus be evaluated carefully.

Our data do not support the often-proclaimed dose-dependent osteoradionecrosis risk increase or any threshold values for the occurrence of osteoradionecrosis [60]. Nevertheless, lower radiation doses might preserve the body from excessive damage, therefore reducing endarteritis and leading to a lesser extent of fragile hypovascular, hypoxic, and hypocellular tissue. Sparing tissue from radiation and placing implants in areas that were exposed to lower radiation doses should be enforced whenever possible.

While we were unable to track the specific location in which ORN appeared, Dumoulin et al.’s (2021) analysis of 32 cases shows that ORN most often occurs in the body of the mandible. The ramus or symphysis was affected less frequently, whereas no case in the maxilla was observed [61].

Analyzing pooled data, implants placed during reconstructive surgery and in grafted bone showed a higher risk for ORN. However, both analyses were severely influenced by the higher number of cases in the article by Fenlon et al. (2012) [59].

In general, using more minimally invasive approaches during secondary implant placement—reducing additional trauma—should be aimed towards to decrease risk. Future prospective trials researching the impact of ideally flapless computer-aided implant surgery (CAIS) on the incidence of osteoradionecrosis could be interesting.

Additional risk factors for ORN that could be isolated by Dumoulin et al. (2021) or Owosho et al. (2017) are smoking, alcohol abuse, diabetes, and poor periodontal status [61, 62].

Shaw et al.’s (2019) randomized controlled trial provided evidence that HBO does not reduce the risk for ORN after oral surgery, which is consistent with our analysis [50].

Pentoxifylline and vitamin E (alpha-tocopherol) have been shown to reduce a patient’s risk of developing osteoradionecrosis after dental extraction [63]. A prophylactic application before implant placement could also be conceivable. There are various other current research projects trying to improve implant osseointegration or soft tissue healing, such as using low-level laser therapy (LLT), the application of bone morphogenetic protein 2 (BMP2), platelet-rich plasma (PRP) treatment, the use of stem cells or optimizing the features of the inserted implants [64–69]. Implementing promising approaches could help to advance implant surgery and reduce the incidence of osteoradionecrosis.

As seen in our study, not only implant surgery but also implant failure or even implant manipulation—such as periimplantitis treatment leading to soft tissue trauma—could cause osteoradionecrosis. Thus, handling those patients carefully, improving implant prognosis and optimal prosthodontic rehabilitation are integral parts of ORN prevention.

Recent publications argue that dental extractions or implantations might not be the cause of ORN but rather accelerate the clinical manifestation of necrotic bone tissue [50, 70, 71]. If this would turn out to be true, reliable screening methods for the preclinical stages of ORN need to be identified and implemented before elective oral surgeries.

### Quality of life and workflow

The goal of placing implants in irradiated head and neck cancer patients is to enable sufficient oral rehabilitation and therefore increase their quality of life while not endangering their survival and staying economically reasonable. However, patients with high morbidity, who would benefit the most from implant surgery, tend to be high-risk patients with a poor long-term prognosis. During the patient selection process, it should be considered if the additional risks, costs, and increase in rehabilitation time are worth the elective surgical procedure to increase the quality of life in high-risk patients with a low prospective 5-year survival. While strict selection is ideal for keeping complication rates low, it also severely limits the number of patients who can benefit from implant surgery.

The percentage of patients who complete treatment (22–91%) and the total time needed for it vary harshly between different studies [72]. Treatment drop-out or long duration negatively impacts patient quality of life.

A well-thought-through treatment concept could help to simplify and optimize the selection process and choose ideal candidates for implant placement, simultaneously reducing treatment time and increasing the number of completed rehabilitations [10, 73]. Criteria for patient selection were defined by Chiapasco et al. (2006), who proposed excluding patients with persisting alcohol or tobacco abuse and poor compliance, whereas good tumor prognosis, sufficient oral hygiene, absence of periodontal disease, or patient request were seen as inclusion criteria [74]. Smolka et al. (2008) evaluated a treatment concept based on the defect classification by Iizuka et al. (2005), in which the number of osteotomies needed to recreate the mandibular contour was the defining factor. In this treatment concept, the mandibular defect size—described by the classification above—dictates the type of prosthodontic rehabilitation. They were able to show a high percentage of successfully completed functional dental reconstructions and had similar results to conventionally planned rehabilitations in terms of implant survival and complication rates [75, 76]. Rouers et al. (2019) proposed an earlier inclusion of the prosthodontist into the treatment process by selecting implant sites before radiation treatment dosimetry [77]. Combining those or similar approaches to standardized workflows could not only potentially impact treatment outcome and the patient’s quality of life but also help to generate more comparable data.

### Limitations

As always, the results of a systematic review should be critically analyzed because there are several limitations. There was an inevitable selection bias because there was a single examiner during study selection. To offset this bias, every doubtful case and every study that was included in the final review were discussed with the co-examiners.

Although most studies are published in English, the exclusion of studies based on their language could contribute to a possible selection bias.

Since cancer patients suffer from a low survival rate, implant placement in irradiated patients is not the norm, and the occurrence of osteoradionecrosis is rare. Large patient groups are necessary to gather reliable information. For the same reasons, the number of prospective or randomized controlled trials is extremely low, and most included studies are retrospective. Thus, we deemed it important to evaluate the quality of each study individually by using the most suitable checklist from The Joanna Briggs Institute for each article.

A side effect of the mostly retrospective study designs was the common lack of control groups. Often, true meta-analysis was not possible, and pooled analysis had to be used when taking a closer look at a specific subgroup. This increases the risk of bias and artificially narrows confidence intervals [78, 79]. There was also a lack of individual data (e.g., on applied radiation dosage), which impeded certain analyses (e.g., radiation dose threshold for implant survival). Furthermore, a relevant difference between the included studies is the definition of implant success or implant survival. Some studies excluded patients who died or had tumor recurrences during the follow-up, while others counted them as successful or saw them as failures. Even if this was accounted for during analysis, the initial data still showed inconsistency.

Additionally, the researched population is quite heterogeneous (e.g., initial diagnosis, defect size, reconstruction method, implant brand and type, prosthesis design, radiation technique, treatment year), and because there are no standardized classification systems in place, comparing individuals in a single clinic—let alone between completely varying settings—is associated with a high risk.

All these factors negatively impact the level of evidence of our systematic review and meta-analysis and show that there is a severe lack of prospective or randomized controlled trials for the oral rehabilitation of head and neck cancer patients.
